# Incidence and Prognosis of Atrial Fibrillation in Patients With Sepsis

**DOI:** 10.4021/cr108w

**Published:** 2011-11-20

**Authors:** Gretchen L. Wells, Peter E. Morris

**Affiliations:** a Department of Internal Medicine-Section on Cardiology, Wake Forest School of Medicine, Medical Center Boulevard, Winston-Salem, NC, USA; b Department of Internal Medicine-Section on Pulmonary/Critical Care, Wake Forest School of Medicine, Medical Center Boulevard, Winston-Salem, NC, USA

**Keywords:** Atrial fibrillation, Sepsis, Mortality, Elderly, Arrhythmia

## Abstract

**Background:**

Although the mortality rate among patients with sepsis is declining, the incidence of both sepsis and sepsis-related deaths is increasing, likely due to its presence in a growing elderly population. As atrial fibrillation is more common in the elderly, we hypothesize that its presence will be associated with greater mortality among patients with sepsis.

**Methods:**

The Medical Intensive Care Unit (MICU) database of a large tertiary care medical center was queried for sepsis-related codes and atrial fibrillation.

**Results:**

Atrial fibrillation was associated with older age and a higher mortality in this series of patients with sepsis.

**Conclusions:**

Whether atrial fibrillation is a marker of disease severity or contributes to mortality is uncertain. Further studies are necessary to determine optimal management.

## Introduction

An emerging challenge in the management of the ICU patient with sepsis is atrial fibrillation. While the current mortality rate among patients with sepsis is declining [1], likely due to improvements in the management of the critically-ill patient including early goal-directed therapy [2], the incidence of sepsis and the number of sepsis-related deaths are increasing, due largely to a growing elderly population [3]. As atrial fibrillation is most common in older patients, we performed a retrospective review over a one-year period of ICU patients hospitalized with sepsis, and we found that atrial fibrillation was not only common, but it was associated with increased mortality.

## Materials and Methods

From January 1, 2008 to December 31, 2008, 1466 patients were admitted to the Medical Intensive Care Unit (MICU) at Wake Forest University Baptist Medical Center, an 885-bed university hospital. All patients admitted to the MICU were queried for sepsis-associated ICD-9 codes (785.52 and 995.92). Patients with a recent myocardial injury during the hospitalization or a malignancy identified by ICD-9 codes were excluded.

## Results

Four hundred sixty-five of these patients were identified with either severe sepsis or septic shock [4]. Of these 465 patients (203 women and 262 men) with a sepsis-associated ICD-9 code, 132 (54 women and 78 men) developed atrial fibrillation identified by telemetry and confirmed by a faculty cardiologist interpretation of an ECG.

The mean age in the ICU population during this period was 63 ± 17 years, and the mean age in the sepsis population was 62 ± 16 years. However, in the patients with both sepsis and atrial fibrillation, the mean age was 72 ± 13 years.

Of the patients with sepsis who developed atrial fibrillation, there was a much higher percentage of coronary artery disease, chronic obstructive pulmonary disease and diabetes mellitus (40%, 39% and 41%, respectively) compared with those patients with sepsis who did not develop atrial fibrillation (19%, 29% and 33%, respectively). However, it appears that these were also risk factors for the development of atrial fibrillation in the general MICU population (i.e. those without sepsis) as well. (Table 1)

**Table 1:** Comparative and Descriptive Analysis of ICU Patients With Sepsis

	Total N = 1466	1	2	3	4	Overall P-value	Odds Ratio (95% Confidence Interval)		
		Sepsis and Afib 132 (9%)	Sepsis only, no Afib 333 (23%)	Afib only, no Sepsis 196 (13%)	Neither 805 (55%)		1 versus 4	2 versus 4	3 versus 4
Mortality	628 (43%)	95 (72%)	189 (57%)	94 (48%)	250 (31%)	< 0.0001	5.7 (3.8, 8.6)	2.9 (2.2, 3.8)	2.0 (1.5, 2.8)
Age (years)	63 ± 17	72 ± 13	62 ± 16	76 ± 11	59 ± 17	< 0.0001^*^			
Gender									
Female	700 (48%)	54 (41%)	149 (45%)	81 (41%)	416 (52%)				
Male	766 (52%)	78 (59%)	184 (55%)	115 (59%)	389 (48%)	0.0079	1.5 (1.1, 2.2)	1.3 (1.02, 1.7)	1.5 (1.1, 2.1)
Race									
Black	384 (26%)	22 (17%)	87 (26%)	36 (18%)	239 (30%)				
Am. Indian	3 (0.2%)	1 (0.8%)	2 (0.6%)	0 (0%)	0 (0%)				
Asian	3 (0.2%)	1 (0.8%)	1 (0.3%)	0 (0%)	1 (0.1%)				
Other	2 (0.1%)	0 (0%)	0 (0%)	1 (0.5%)	1 (0.1%)				
Hispanic	22 (1.5%)	0 (0%)	7 (2%)	0 (0%)	15 (1.9%)				
Unknown	18 (1.2%)	2 (1.5%)	5 (1.5%)	1 (0.5%0	10 (1.2%)				
White	1034 (71%)	106 (80%)	231 (69%)	158 (81%)	539 (67%)				
Race2									
White	1034 (71%)	106 (80%)	231 (69%)	158 (81%)	539 (67%)	0.0001	2.0 (1.3, 3.2)	1.1 (0.8, 1.5)	2.0 (1.4, 3.0)
Non-White	432 (29%)	26 (20%)	102 (31%)	3 (19%)	266 (33%)				
Underlying Diseases									
CAD	351 (24%)	53 (40%)	64 (19%)	73 (37%)	161 (20%)	< 0.0001	2.7 (1.8, 4.0)	0.95 (0.7, 1.3)	2.4 (1.7, 3.3)
COPD	476 (32%)	52 (39%)	96 (29%)	69 (35%)	259 (32%)	0.1328			
Diabetes	513 (35%)	54 (41%)	111 (33%)	73 (37%)	275 (34%)	0.3741			

The table above includes p-values for the overall comparison of any difference between the four groups. When the overall comparison is statistically significant, the odds ratios or mean comparisons are given (depending on the variable type).

For the binary variables (yes/no, male/female, white/non-white, death/no death), Odds Ratios and the 95% Confidence Interval are given with group 4 (Neither) as the reference group. An odds ratio of 1.0 indicates no difference between the group being evaluated and the reference group. Therefore, if the 95% confidence interval includes one, there is no difference between the two groups.

Interpretation example: There is a significant difference in mortality between the four groups. Group 1 is 5.7 times more likely to die if they are in the Sepsis and Afib group compared to the Neither group.

For age, the continuous variable, multiple comparisons of the mean age between the four groups was done with a Bonferroni correction. There was a significant difference between all groups except 1 versus 3.

Atrial fibrillation was strongly associated with in-hospital mortality in the MICU patient (P < 0.001). Of the patients without sepsis who developed atrial fibrillation, 94 of these 196 individuals died (48%). Even more striking is the mortality in the group with sepsis who developed atrial fibrillation where 95 of 132 (72%) patients died. (Table 2)

**Table 2:** Comparative and Descriptive Analysis of ICU Patients With Sepsis and Death (Descriptives for Deaths Only)

	Total N = 628	1	2	3	4	Overall P-value	Odds Ratio (95% Confidence Interval)		
		Sepsis and Afib, N = 95	Sepsis only, no Afib, N = 189	Afib only, no Sepsis, N = 94	Neither N = 250		1 versus 4	2 versus 4	3 versus 4
Age (years)	68 ± 15	73 ± 13	65 ± 15	78 ± 10	66 ± 14	< 0.0001			
Gender									
Female	278 (44%)	36 (38%)	83 (44%)	43 (46%)	116 (46%)				
Male	350 (56%)	59 (62%)	106 (56%)	51 (54%)	134 (54%)	0.5504			
Race									
Black	158 (25%)	19 (20%)	51 (27%)	19 (20%)	69 (28%)				
Am. Indian	3 (0.5%)	1 (1%)	2 (1%)	0 (0%)	0 (0%)				
Asian	2 (0.3%)	1 (1%)	0 (0%)	0 (0%)	1 (0.4%)				
Other	1 (0.16%)	0 (0%)	0 (0%)	0 (0%)	1 (0.4%)				
Hispanic	6 (1.0%)	0 (0%)	2 (1%)	0 (0%)	4 (1.6%)				
Unknown	9 (1.4%)	1 (1%)	3 (1.6%)	1 (1%)	4 (1.6%)				
White	449 (72%)	73 (77%)	131 (69%)	74 (79%)	171 (68%)				
Race2									
White	449 (72%)	73 (77%)	131 (69%)	74 (79%)	171 (68%)	0.1473			
Non-White	179 (28%)	22 (23%)	58 (31%)	20 (21%)	79 (32%)				
Underlying Diseases									
CAD	453 (72%)	41 (43%)	41 (22%)	38 (40%)	55 (22%)	< 0.0001	2.7 (1.6, 4.4)	0.98 (0.6, 1.5)	2.4 (1.4, 4.0)
COPD	211 (34%)	35 (37%)	57 (30%)	34 (36%)	85 (34%)	0.6265			
Diabetes	224 (36%)	41 (43%)	61 (32%)	36 (38%)	86 (34%)	0.2922			

The table above includes p-values for the overall comparison of any difference between the four groups. When the overall comparison is statistically significant, the odds ratios or mean comparisons are given (depending on the variable type).

For the binary variable-CAD, Odds Ratios and the 95% Confidence Interval are given with group 4 (Neither) as the reference group. An odds ratio of 1.0 indicates no difference between the group being evaluated and the reference group. Therefore, if the 95% confidence interval includes one, there is no difference between the two groups.

For age, the continuous variable, multiple comparisons of the mean age between the four groups was done with a Bonferroni correction. There was a significant difference between all groups except 1 versus 3.

## Discussion

Critically-ill patients frequently develop cardiac arrhythmias (up to 90% in primary cardiovascular patients); however, these groups often include those patients post major surgery, multiple trauma, those with severe underlying lung disease, malignancies, renal failure, and neurologic diseases as well as sepsis [5, 6]. Only one report described 25 of 81 patients admitted with sepsis (31%) developing paroxysmal atrial fibrillation [7]. This finding is similar to that of ours in which 132 out of 333 patients with sepsis (40%) developed atrial fibrillation.

Cardiac involvement occurs in septic patients, even without septic shock. The ejection fraction (fraction of end-diastolic volume ejected with each beat) is often reduced, likely through several mechanisms including inflammatory mediators [8]. It is plausible that the cardiac involvement would include the development of atrial fibrillation. However, one study did not link inflammation (identified by C-reactive protein levels) with the development of post-operative atrial fibrillation [9].

Early goal-directed therapy of sepsis was designed to optimize cardiac preload, afterload, and contractility and was associated with a significant mortality benefit. However, large-volume infusion results in less of an increase in left ventricular stroke work index in patients with sepsis and septic shock as compared to other critically-ill control subjects [10]. Whether large volume infusion reduces or increases atrial arrhythmias is unknown.

The prevalence of atrial fibrillation is estimated to be 0.4 to 1% of the general population, increasing with age to > 8% in those over 80 years of age. It is complicated by an increased risk of stroke, heart failure and mortality. Anticoagulation, rate control and rhythm control strategies are treatment options; however, large studies have not demonstrated that rhythm control is superior to rate control in certain populations [11]. Some studies have focused on the prevention of atrial fibrillation, e.g., amiodarone, angiotensin-converting enzyme inhibitors and 3-hydroxy-3-methylglutaryl coenzyme A reductase inhibitors (statins) with limited success [12]. Finally, catheter ablation for paroxysmal atrial fibrillation has emerged as an effective treatment in selected patients [13].

Guidelines and strategies for the acute and chronic management of atrial fibrillation are available for many populations [14, 15]. However, after an exhaustive review of the literature, Kanji et al. could not recommend a treatment strategy for atrial fibrillation in the noncardiac, critically-ill adult patient due to a lack of clinical trials) [16]. Management decisions are even more complicated among typical ICU patients who are elderly, have multiple comorbidities, and have been excluded from clinical trials of atrial fibrillation [17].

Our series, as well as others, demonstrate that atrial fibrillation frequently complicates the course of sepsis and results in increased mortality [5, 7, 18-20], although one series reported no impact on the risk of in-hospital mortality [6]. Whether this arrhythmia is a marker of critical illness or the cause of death is uncertain. Further studies are warranted to determine the optimal management of atrial fibrillation in this older, high-risk, critically-ill population.
